# Differences between human and chimpanzee genomes and their implications in gene expression, protein functions and biochemical properties of the two species

**DOI:** 10.1186/s12864-020-06962-8

**Published:** 2020-09-10

**Authors:** Maria V. Suntsova, Anton A. Buzdin

**Affiliations:** 1 Institute for personalized medicine, I.M. Sechenov First Moscow State Medical University, Trubetskaya 8, Moscow, Russia; 2 Shemyakin-Ovchinnikov Institute of Bioorganic Chemistry of the Russian Academy of Sciences, Miklukho-Maklaya, 16/10, Moscow, Russia; 3 Omicsway Corp, Walnut, CA USA; 4 Moscow Institute of Physics and Technology (National Research University), 141700 Moscow, Russia

**Keywords:** Human-specific, Chimpanzee, Genome alterations, Genetic differences, Molecular evolution

## Abstract

Chimpanzees are the closest living relatives of humans. The divergence between human and chimpanzee ancestors dates to approximately 6,5–7,5 million years ago. Genetic features distinguishing us from chimpanzees and making us humans are still of a great interest. After divergence of their ancestor lineages, human and chimpanzee genomes underwent multiple changes including single nucleotide substitutions, deletions and duplications of DNA fragments of different size, insertion of transposable elements and chromosomal rearrangements. Human-specific single nucleotide alterations constituted 1.23% of human DNA, whereas more extended deletions and insertions cover ~ 3% of our genome. Moreover, much higher proportion is made by differential chromosomal inversions and translocations comprising several megabase-long regions or even whole chromosomes. However, despite of extensive knowledge of structural genomic changes accompanying human evolution we still cannot identify with certainty the causative genes of human identity. Most structural gene-influential changes happened at the level of expression regulation, which in turn provoked larger alterations of interactome gene regulation networks. In this review, we summarized the available information about genetic differences between humans and chimpanzees and their potential functional impacts on differential molecular, anatomical, physiological and cognitive peculiarities of these species.

## Background

The divergence of human and chimpanzee ancestors dates back to approximately 6,5–7,5 million years ago [1] or even earlier [2]. It is still of a great interest to identify genetic elements that distinguish humans from chimpanzees and encode features of human physiological and mental identities [3–5]. It’s a difficult task to quantitate the exact percentage of differences between human and chimpanzee genomes. In early works, divergence of human and chimpanzee genomes was estimated as roughly 1% [6]. This estimate was based on the comparison of protein-coding sequences and didn’t consider non-coding (major) part of DNA. However, the idea of ~ 99% similarity of genomes persisted for a long time, until 2005 when nearly complete initial sequencing results of both human [7] and chimpanzee (*Pan troglodytes*) [8] genomes became available. It was found that genome differences represented by single nucleotide alterations formed 1.23% of human DNA, whereas larger deletions and insertions constituted ~ 3% of our genome [8]. Moreover, even higher proportion was shaped by chromosomal inversions and translocations comprising several megabase-long chromosomal regions or even entire chromosomes, as for the chromosomal fusion that took place when the human *chromosome 2* was formed [9]. Here we tried to review the major known structural and regulatory genetic alterations that had or might have a functional impact on the human and chimpanzee speciation (Table 1).

**Table 1:** Molecular genetic differences between humans and chimpanzees

Human	Chimpanzee
**Karyotype**	
46 chromosomes. Chromosome 2 was formed by fusion of two ancestral chromosomes [9].	48 chromosomes, including chromosomes 2a and 2b [9].
Large pericentric inversions in chromosomes 1 and 18 [10–12].	Large pericentric inversions in chromosomes 4, 5, 9, 12, 15–17 [10–12].
Two pseudoautosomal regions, PAR2 and PAR3 on Y chromosome [13–15].	
Different amounts of pericentric, paracentric, intercalary and Y type heterochromatin [9].	
**Deletions, insertions, copy number variations (~ 3% of genomes differences)**	
Several hundreds of species-specific processed pseudogenes [8, 16].	
134 genes increased copy numbers, 6 decreased [17]. *SRGAP2* duplicated with the formation of two truncated homologs *SRGAP2B* and *SRGAP2C* [18].	37 genes increased copy numbers, 15 – decreased [17].
Deletion of 510 conserved regions. Among them: androgen receptor (AR) enhancer, tumor suppressor GADD45G enhancer, CMAHP exon, etc. [19, 20]. Human-specific mobile elements recombination/insertion-associated deletions: at least 492 Alu-associated deletions(~ 400 kb of excised DNA) [21], at least 73 LINE-associated deletions (~ 450 kb of excised DNA) [22, 23], at least 26 SVA-associated deletions (~ 46 kb of excised DNA) [24].	Deletion of 334 conserved regions [19]. Chimpanzee-specific mobile elements recombination/insertion- associated deletions: at least 663 Alu-associated deletions(~ 771 kb of excised DNA) [25].
Mobile elements	
• Alu: ~ 5000 unique copies, AluYa5 и AluYb8 families prevail [8, 26, 27] • LINE L1: ≥ 2000 of unique insertions [26, 28] • SVA (SINE-VNTR-Alu): several thousands of specific insertions, two times more active retrotransposition [26, 27]. New family emerged - CpG-SVA or SVAF1 [29, 30]. • HERVs: ~ 140 unique insertions of HERV-K (HML-2) [31–34]. Several hundred copies of HERV-K (HML-2) К111 and several dozen copies of HERV-K (HML-2) K222 emerged due to recombination in centromeric and pericentromeric regions [35, 36].	• Alu: ~ 1500 unique copies, Alu Y and Yc1 families prevail [8, 26, 27] • LINE L1: ≥ 2000 of unique insertions [26, 28] • SVA: several thousands of specific insertions [26, 27] • HERVs: ~ 45 unique insertions of HERV-K (HML-2) [37, 38] • Two new families emerged – PtERV1 and PtERV2 (totally around 250 copies) [8, 39]
**Single nucleotide alterations (substitutions, insertions, deletions): ~ 1.23% of genomes differences**	
Protein-coding sequences	
• Different repertoires of olfactory receptor genes and pseudogenes, 25% out of ~ 400 active genes are species-specific [40]. • Highly diverged genes relate to immunity and cell recognition. Point mutations inactivated genes of T-cell gamma-receptor TCRGV10, caspase 12, mannose-binding lectin gene MBL1P, etc. [8, 41, 42]. • Species-specific mutations in genes responsible for sialic acids metabolism: *ST6GAL1*, *ST6GALNAC3*, *ST6GALNAC4*, *ST8SIA2*, *HF1*. Point mutations in genes *SIGLEC11* and *SIGLEC12* abrogated their sialic-binding activities [8, 43–46] • Substitutions in language-associated gene *FOXP2*: Thr303Asn and Asn325Ser [43, 44]. • Quickly evolving brain size-related genes *MCPH1* and *ASPM* [46, 47].	• Different repertoires of olfactory receptor genes and pseudogenes, 25% out of ~ 400 active genes are species-specific [40]. • Highly diverged genes relate to immunity and cell recognition [8, 41, 42] • Species-specific mutations in genes responsible for sialic acids metabolism: *ST6GAL1, ST6GALNAC3, ST6GALNAC4, ST8SIA2, HF1* [8]
Non-coding sequences	
~ 3000 of human accelerated regions: HARs and HACNs [48, 49]. HARS and HACNs are enriched in genes related to DNA interaction, transcriptional regulation and neuronal development [50]. *NPAS3* (neuronal PAS domain-containing protein) gene contains 14 HARs. The most rapidly evolving regions HAR1 and HARE5 are located in brain-related genes: *HAR1F/HAR1R*-overlap and *FZD8* [48, 49, 51]. ~ 100 of human-specific enhancers activated in nervous tissues (hEANTs) [52]	

### Karyotype

Human karyotype is represented by 46 chromosomes, whereas chimpanzees have 48 chromosomes [9]. In general, both karyotypes are very similar. However, there is a major difference corresponding to the human *chromosome 2*. It has originated due to a fusion of two ancestral acrocentric chromosomes corresponding to *chromosomes 2a* and *2b* in chimpanzee. Also, significant pericentric inversions were found in nine other chromosomes [9]. Two out of nine are thought to occur in human *chromosomes 1* and *18*, and the other seven – in chimpanzee *chromosomes 4, 5, 9, 12, 15, 16* and *17* [10–12]. In addition, there are numerous differences in the chromosomal organization of pericentric, paracentric, intercalary and Y type heterochromatin; for example, the chimpanzees have large additional telomeric heterochromatin region on *chromosome 18* [9]. Additionally, the majority of chimpanzee’s chromosomes contain subterminal constitutive heterochromatin (C-band) blocks (SCBs) that are absent in human chromosomes. SCBs predominantly consist of the subterminal satellite (StSat) repeats, they are found in African great apes but not in humans [53]. The presence of such SCBs affects chimpanzees’ chromosomes behavior during meiosis causing persistent subtelomeric associations between homologous and non-homologous chromosomes. As a result of homologous and ectopic recombinations chimpanzees demonstrate greater chromatin variability in their subtelomeric regions [54].

Studying sex chromosomes also revealed several peculiar traits. There are several regions of homology between *X* and *Y chromosomes*, so-called pseudoautosomal regions (PARs) most probably arisen due to translocation of DNA from *X* to *Y chromosome* [13]. The term “pseudoautosomal” means that they can act as autosomes being involved in recombination between *X* and *Y chromosomes*. PAR1 is a 2,6 Mb long region located at the end of *Y chromosome* short arm. It is homologous to the terminal region of the short arm on *X chromosome*. PAR2 is a 330 kb-long sequence located on the termini of long arms of *X* and *Y chromosomes*. In contrast to PAR1 presenting in many mammalian genomes, PAR2 is human-specific [14]. It includes four genes: *SPRY3, SYBL1, IL9R* and *CXYorf1. The first two genes (SPRY3, SYBL1) are silent on the Y chromosome (SPRY3, SYBL1) and are subjects of X-inactivation-like mechanism. On the other hand, the genes IL9R and CXYorf1 are active in both sex chromosomes* [55, 56]. Moreover, the short arm of *Y* contains a 4 Mb-long translocated region from the long arm of *X chromosome*, called X-translocated region (XTR) [14, 57]. A part of the XTR has undergone inversion due to recombination between the two mobile elements of LINE-1 family. Both translocation and inversion took place already after separation of human and chimpanzee ancestors [14, 58]. Finally, this region also includes genes *PCDH11Y* and *TGIF2LY* which correspond to *X chromosome* genes *PCDH11X* and *TGIF2LX* [15]. Around 2% of human population have signs of recombination between *X* and *Y chromosomes* at the XTR. It should be considered, therefore, as an additional human-specific pseudoautosomal region PAR3 [15].

### Insertions, deletions and copy number variations

Enzymatic machinery of LINE1 retrotransposons not only reverse transcribes its own RNA molecules, but also frequently produces cDNA copies of other cellular transcripts, e.g. host genes or non-coding RNAs [59, 60] Sometimes a template switch can occur due to reverse transcription, thus resulting in double or even triple chimeric retrotranscripts [61]. Reverse-transcribed copies of the host genes are called processed pseudogenes [62]. Immediately after primary assembly of the human and chimpanzee genomes, nearly 200 human- and 300 – chimpanzee-specific processed pseudogenes were identified. Most of them were copies of ribosomal protein genes which accounted for ~ 20% of species-specific pseudogenes [8]. However, these numbers were significantly underestimated. For example, another study revealed already ~ 1800 and 1500 processed pseudogenes of ribosomal protein in the human and chimpanzee genomes, respectively, of which ~ 1300 were common [16].

In addition to genome sequencing, DNA hybridization arrays were widely used for copy number variation studies [63, 64]. In human, microarray assay revealed a relatively increased copy number of 134 and decreased - of six genes compared to the genomes of other great apes such as chimpanzee (*Pan troglodytes*), bonobo (*Pan paniscus*), gorilla (*Gorilla gorilla*) and orangutan (*Pongo pygmaeus*) [17]. However, the figure of six genes with decreased copy numbers was certainly an underestimation because hybridization was performed using the probes for human genes. This assay also couldn’t distinguish functional genes and pseudogenes. Anyway, the human-amplified group was found to be enriched in genes involved in central nervous system (CNS) functioning. These were *NAIP* (neuronal apoptosis inhibitory protein), *SLC6A13* (gamma-aminobutyric acid (GABA) transporter), *KIAAA0738* (zinc-finger transcription factor, expressed in brain), *CHRFAM7A* (fusion of acetylcholine receptor gene and *FAM7*), *ARHGEF5* (guanine exchange factor), *ROCK1* (Rho-dependent protein kinase), and also members of the gene families: *ARHGEF*, *PAK, RhoGAP* and *USP10* (ubiquitin-specific protease) associated with various forms of mental retardation. Relatively to humans, chimpanzees had increased copy numbers of 37 and decreased copy numbers of 15 genes [17].

The same study also revealed increased copy number of Rho GTPase-activating protein *SRGAP2* gene in human genome [17]. There were also two truncated human-specific homologs of this gene: *SRGAP2B* and *SRGAP2C.* The experiments with mouse embryos showed that *SRGAP2* could facilitate maturation and limit density of dendrite spines in the developing neurons in neocortex. Truncated protein SRGAP2C forms a dimeric complex with the normal SRGAP2 and inhibits it. Apparently, physiological expression of *SRGAP2C* and *SRGAP2B* could impact human brain development by causing specific increase of spine density and extension of maturation of pyramidal neurons in human neocortex [18].

Another study was focused on sequences conserved in chimpanzees and other primates but underrepresented in humans (termed hCONDELs) [19]. Comparison of human, chimpanzee and macaque genomes revealed 510 conserved regions deleted in humans, all of them representing non-coding sequences except *CMAHP* gene, see below. The hCONDELs identified were enriched near genes involved in steroid hormone signaling and neuronal functioning. One hCONDEL was a sensory vibrissae and penile spines-specific enhancer of androgen receptor (*AR*) gene. Its absence caused the loss of vibrissae and spines in humans. Another deletion involved enhancer of a tumor suppressor gene *GADD45G*, which activated expression of this gene in the subventricular zone of the forebrain. It could relate to the specific pattern of expansion of brain regions in humans. In turn, the chimpanzee genome also lacks several conserved sequences. Among 344 such regions identified, significant enrichment was found for the localizations near genes related to synapse formation and functioning of glutamate receptors [19].

Finally, substantial differences in copy numbers were reported for transposable elements (TEs). According to various estimates, the number of human-specific TE insertions varied from eight [26] to 15,000 copies [27]. It was estimated that humans have approximately twice as many unique TE copies as the chimpanzees [8, 26]. Since human-chimpanzee ancestral divergence, the most active TE groups were *Alu, LINE1* and *SVA* which accounted for nearly 95% of all species-specific insertions [26]. The most numerous group was *Alu*, which made over 5 thousand human-specific insertions and proliferated approx. Three times more intensely in humans than in chimpanzees [26, 27]. Most of chimpanzee-specific *Alu* copies are represented by subfamilies *Alu Y* and *AluYc1*, while human-specific insertions are predominantly the members of *AluYa5* and *AluYb8* subfamilies [8, 26]. However, both species also have specific inserts of *AluS* and *AluYg6* family members.

Besides insertional polymorphism, *Alu* also impacted divergence of the two genomes through homologous recombination. At least 492 human-specific deletions emerged because of recombinations between the different *Alu* copies that made ~ 400 kb of excised DNA. Of them, 295 deletions covered known or predicted genes [21]. For example, the aforementioned *CMAHP* gene lost its 6th exon due to recombination event between the two *Alu* elements [20]. Another example is tropoelastin gene. In most vertebrates, it has 36 exons. During the evolution, primate ancestors have lost the 35th exon, and then human ancestors additionally lost the exon 34, also most probably due to recombination between the *Alu* elements [65]. On the other hand, *Alu-Alu* recombinations had significant impact also for the chimpanzee genome: at least 663 such chimpanzee-specific deletions lead to 771 kb DNA loss, and roughly a half of them took place inside gene regions [25].

The activities of *LINE-1* transposable elements were comparable in humans and chimpanzees and resulted in over 2000 species-specific integrations [28]. *LINE-1* is ~ 6 kb-long TE harboring two open reading frames. The majority of *LINE-1* inserts are 5′-truncated, most probably due to apparently abortive reverse transcription [66]. Interestingly, among the human-specific TEs there were several times more full-length *LINE-1* elements with intact open reading frames. The species-specific insertions were made by the members of the *LINE-1* subfamilies *L1-Hs* and *L1-PA2* [26, 28, 67]. In addition, *LINE-1* elements were responsible for at least 73 human-specific deletions collectively resulting in a loss of nearly 450 kb of genomic DNA [22, 23].

Another family termed *SVA* (SINE-VNTR-Alu) elements is represented in the human genome by about 1000 species-specific genomic copies, which is approximately twice higher than in the chimpanzee [26, 27]. Noteworthy, the human genome contains at least 84 insertions of a new, exclusively human-specific type of transposable elements called *CpG-SVA* or *SVAF1*, formed by CpG-island of human gene *MAST2* fused with 5′-truncated fragment of *SVA*. This group most likely emerged through insertion of an SVA element into the first exon of *MAST2* gene containing a CpG-island. Because of *MAST2* promoter activity, a chimeric transcript was formed, processed and then reverse transcribed by *LINE-1* enzymatic machinery followed by insertions into a plethora of new genomic positions. For these new copies of a hybrid element, *MAST2* CpG island enabled male germ line-specific expression, thus facilitating fixation in the genome [29, 30]. Finally, like the other major groups of TEs *SVA* elements also mediated loss of human genomic DNA. At least 26 cases of SVA-associated human-specific deletions were mentioned in the literature, which totally resulted in ~ 46 kb of deleted DNA [24].

After split of human and chimpanzee ancestors, there was also a *HERV-K (HML-2)* family of endogenous retroviruses that was proliferating in both genomes [31, 32, 68, 69]. Its insertional activity resulted in ~ 140 human-specific copies that formed ~ 330 kb of human DNA [31–34], some of them being polymorphic in human populations [69–74]. In turn, the chimpanzee genome has at least 45 species-specific insertions of these elements [37, 38]. In addition, two new specific retroviral families – *PtERV1* and *PtERV2* with 250 totally chimpanzee-specific copies, arose already in the chimpanzee genome [8, 39].

The new copies of transposable elements can appear in the genome not only through insertions but also due to duplications of genomic DNA. For example, several hundred copies of recently integrated *HERV-K (HML-2)* family provirus *К111* were found in centromeres of 15 different human chromosomes. They amplified and spread due to recombinations of the enclosing progenitor locus. In contrast, there is only one copy of *К111* in the chimpanzee genome and no copies in the other primates [35, 36]. Similarly, several dozen copies of a more ancient provirus *K222* of the same family arose due to chromosomal recombination in pericentromeric regions of nine human chromosomes, versus only one copy in the chimpanzees and other higher primates [36].

Furthermore, a human-specific endogenous retroviral (ERV) insert was demonstrated to serve as the tissue-specific enhancer driving hippocampal expression of *PRODH* gene responsible for proline degradation and metabolism of neuromediators in CNS [75]. Finally, the ERVs can provide their promoters for expression of non-coding RNAs from the downstream genomic loci [76]. Almost all ERV inserts in introns of human genes were fixed in the antisense orientation relative to gene transcriptional direction [77], most probably because of the interference of gene expression with their polyadenylation signals. However, it has a functional consequence of ERV-driven antisense transcripts overlapping with human genes. For two genes, *SLC4A8* (for sodium bicarbonate cotransporter) and *IFT172* (for intraflagellar transport protein 172), these human-specific antisense transcripts overlap with the exons and regulate their expression by specifically decreasing their mRNA levels [78].

TE inserts also could play an important role in the speciation. TEs contain various regulatory elements such as promoters, enhancers, splice-sites and signals of transcriptional termination, which they use for their own expression and spread. Approximately 34% of all species-specific TEs in humans and chimpanzees are located close to known genes [26]. Species-specific TE inserts, therefore, can strongly influence regulatory landscape of the host genome [79, 80]. In addition, TEs can disrupt gene structures by inserting themselves or through recombinations between their copies [21, 23]. These events could influence gene functioning and might cause the respective phenotypic differences [81, 82].

It is worth to note that the main complication of the earlier studies was connected with the quality of non-human genomes assembly. First of all, there were persisting several thousand gaps in the chimpanzee genome, which made a substantial fraction of DNA inaccessible for comparisons. Second, the final stages of apes genomes assemblies and annotations were performed using the human genome as a template [8]. This obviously bias results by “humanizing” great ape genomes thereby concealing some human-specific structural variations. The combination of long-read sequence assembly and full-length cDNA sequencing for de novo chimpanzee genome assembly without guidance from the human genome allowed to overcome this problem [83]. Comparison of de novo sequenced and independently assembled human and great ape genomes revealed 17,789 fixed human-specific structural variants (fhSVs), including 11,897 fixed human-specific insertions and 5892 fixed human-specific deletions. Among fhSVs, a loss of 13 start codons, 16 stop codons, and 61 exonic deletions in the human lineage were detected. Also, fhSVs affected 643 regulatory regions near 479 genes. Totally, 46 fhSVs deletions were detected that were expected to disrupt human genes, 41 of them were new. The affected genes included for example caspase recruitment domain family member 8 (CARD8), genes FADS1 and FADS2 involved in fatty acids biosynthesis, and two cell cycle genes WEE1 and CDC25C [83].

### Single nucleotide alterations

Human specific single nucleotide alterations constitute ~ 1.23% of our genome. This value was found by directly comparing human with chimpanzee genomes. It was very close to the previous theoretical estimate of 1.2% calculated using average divergence rate for autosomes, for the time of human and chimpanzee ancestor’s divergence [84]. In the human populations, ~ 86% of all human specific single nucleotide alterations is fixed and the rest 14% is polymorphic [8]. Remarkably, the lowest and the highest human-chimpanzee nucleotide sequence divergences, 1.0 and 1.9%, respectively, were detected in the chromosomes X and Y. Outstandingly, as much as 15% of all ancestral CG-dinucleotides underwent mutations either in the human or in the chimpanzee lineage [85].

### Protein-coding sequences

Protein coding sequences are 99.1% identical between the two species [86], and in two-thirds of the proteins amino acid sequences are absolutely the same [8]. Generally, in comparison with the model of the latest common ancestor genome, the chimpanzee has more genes that underwent positive selection than human. This can be explained by the different effective sizes of ancestral populations of the two species [87]. However, after divergence, transcription factors (TFs) were the fastest evolving group of genes, and human TFs had ~ 1,5 times higher amino acids substitution rate [8]. Second, genes linked with neuronal functioning also evolved faster in the human lineage [88].

There is a connection identified between mutations in the transcription factor *FOXP2* gene and speech disorders, and an assumption was made that *FOXP2* is responsible for speech and language development in humans. Indeed, the sequence analysis revealed that *FOXP2* has signs of positive selection during human evolution [43] having two human-specific amino acid substitutions: Thr303Asn and Asn325Ser, where the latter led to a new potential phosphorylation site [44]. In vivo experiments showed that these substitutions may have important functional significance. Transgenic mice with humanized version of their *FoxP2* gene demonstrated faster learning when both declarative and procedural mechanisms were involved. Also, they had peculiar dopamine levels and higher neuronal plasticity in the striatum [45].

The microcephalin gene *MCPH1* is involved in the regulation of brain development. Its mutations are linked with severe genetic disorders like microcephaly. During human speciation, this gene evolved under strong positive selection, which is still going on in the modern human population [46]. Another gene connected with the brain size regulation, *ASPM* (abnormal spindle-like microcephaly associated, *MCPH5*), also evolved faster in hominids than in the other primates, having the highest rate of non-synonymous to synonymous substitutions in the human lineage [47].

Several sexual reproduction genes were also among the most rapidly evolving and positively selected hits [44, 89], such as protamine genes *PRM1* and *PRM2* encoding histone analogs in sperm cells. Remarkably, human protamines evolve oppositely to histones, whose structures are highly conservative [89].

Another group of highly diverged genes relates to immunity and cell recognition [8]. A point mutation in the variable domain of T-cell gamma-receptor TCRGV10 destroyed a donor splice-site, which prevented splicing of the leader intron. Chimpanzees don’t have this mutation and their gene remains functional [41].

Both species have many specific mutations in the genes involved in sialic acids metabolism - *ST6GAL1, ST6GALNAC3, ST6GALNAC4, ST8SIA2* and *HF1* [8]. Sialic acids, or N-acetyl neuraminic (Neu5Ac) and N-glycolyl neuraminic acid (Neu5Gc), are common components of the carbohydrate cell surface complexes in mammals. Humans are exceptional because they completely lack Neu5Gc on their cell surfaces [90] because their gene *CMAHP* coding an enzyme – cytidine monophosphate-N-acetylneuraminic acid hydroxylase – responsible for the conversion of CMP-Neu5Ac into CMP-Neu5Gc, has lost its activity. It happened because of the loss of a 92-nucleotide exon corresponding to the sixth ancestral exon, caused by insertion of an AluY element followed by recombination [20, 91].

Moreover, the mechanism of sialic acids recognition was also affected in the human lineage. Human gene *SIGLEC11* for sialic acid receptor underwent a conversion with the pseudogene *SIGLEC16* that significantly compromised its ability to bind sialic acids. However, it still can bind oligosialic acids (Neu5Acα2–8)_2–3_, that are highly abundant in the brain. Moreover, *SIGLEC11* demonstrates human-specific expression in microglia [92]. Similarly, the protein SIGLEC12 lost its sialic acid-binding activity due to human-specific substitution R122C. Nevertheless, *SIGLEC12* gene is still expressed in macrophages and in several epithelial cell types [93].

Another major affected group of genes is for the olfactory receptors. Humans and chimpanzees have a comparable number of olfactory receptor genes, around 800, and 689 of them are orthologous in the two species [40]. However, in both species about half of them have lost their activities and became pseudogenes. Even though the final numbers of active genes are equal in human and chimpanzee, their repertoire is strikingly different – as much as 25% of the active olfactory receptor genes are species-specific. This has led to an assumption that the most recent common ancestor had more active olfactory receptor genes than modern humans and chimpanzees [40].

Other examples include caspase 12, mannose-binding lectin gene *MBL1P* and keratin isoform *KRTHAP1* that lost their activities due to human-specific mutations [8, 42, 94].

### Non-coding sequences

Non-coding sequences play crucial roles in gene regulation [95, 96]. Analysis of species-specific polymorphisms revealed that 96% of regions with the *highest density of alterations* (HAR, human accelerated region) map on non-coding DNA. The genes located near HARs are predominantly related to interaction with DNA, transcriptional regulation and neuronal development [48, 97].

The biggest number of HARs was observed for the *NPAS3* (neuronal PAS domain-containing protein) gene. It codes for a transcription factor involved in brain development. The 14 HARs_*NPAS3*_ are located in non-coding regions and most of them may have regulatory functions, as confirmed by enhancer activities demonstrated in cell culture assay [98].

Rapidly evolving human genome region HAR1 was found in the overlap of two non-coding RNA genes: *HAR1F* and *HAR1R.* The former is expressed at 7–19 weeks of embryonic development in the Cajal-Retzius cells of the emerging neocortex. At the later gestation period and in adulthood *HAR1F* is expressed also in the other parts of the brain. This expression pattern is conserved in all higher primates, but human-specific nucleotide alterations affected the secondary structure of this RNA [48, 99]. Another accelerated region HARE5 (HAR enhancer 5) is ~ 1,2 kb long enhancer of *FZD8* gene. After human and chimpanzee ancestral divergence, their orthologous loci accumulated 10 and 6 nucleotide substitutions, respectively. *FZD8* encodes a receptor protein in the WNT signaling pathway, which is involved in the regulation of brain development and size. In mouse, endogenous HARE5 homolog physically interacts with *Fzd8* core promoter in the neocortex. In transgenic mice with *Fzd8* under control of either human or chimpanzee enhancer, both demonstrated their activities in the developing neocortex, but the human enhancer became active at the earlier stages of development and its effect was more pronounced. Embryos with the human HARE5, therefore, showed a marked acceleration of neural progenitor cell cycle and increased brain size [51].

There is also a particular fraction of non-coding sequences that was accelerated in humans but relatively conserved in the other species called HACNs (human accelerated conserved noncoding sequences) [49]. They can overlap with the abovementioned HARs [50]. HACNs are enriched near genes related to neuronal functioning, such as neuronal cell adhesion [49] and brain development [100]. Based on structural analyses of HACNs, HARs and their genomic contexts, around one third of them was predicted to be developmental enhancers [50]. By functional role, they contribute in approximately equal proportions to brain and limb development and to a lesser extent - to heart development. Among 29 pairs of HARs and their chimpanzee orthologous regions tested in mouse embryos, 24 showed enhancer activity in vivo. Moreover, five of them demonstrated differential enhancer activities between human and chimpanzee sequences [50].

In another study, all human enhancers predicted by the FANTOM project [101] were aligned with the primate genomes in order to obtain human-specific fraction [52]. Notably, the fastest evolving human enhancers predominantly regulated genes activated in neurons and neuronal stem cells. Totally, about 100 human-specific neuronal enhancers were identified, and one of them located on the 8q23.1 region was presumably related to Alzheimer’s disease development. It was assumed by the authors that recent human-specific enhancers, adaptive, on the one hand, may also impact age-related diseases [52].

### Transcriptional regulation

It has been postulated few decades ago that differences between humans and chimpanzees are mostly caused by gene regulation changes rather than by alterations in their protein-coding sequences, and that these changes must affect embryo development [6]. For example, evolutional acquisitions such as enlarged brain or modified arm emerged as a result of developmental changes during embryogenesis [102, 103]. Such changes include when, where and how genes are expressed. A plethora of genes involved in embryogenesis have pleiotropic effects [104] and mutations within their coding sequence may cause complex, mostly negative, consequences for an organism. On the other hand, changes in gene regulation could be limited to a certain tissue or time frame that can enable fine tuning of a gene activity [105]. Indeed, the fast-evolving sequences (HARs or HACNs) are often found close to the genes active during embryo- and neurogenesis [48–50, 100]. For example, HACNS1 (HAR2) demonstrates greater enhancer activity in limb buds of transgenic mice compared to orthologous sequences from chimpanzee or rhesus macaque [106]. A similar pattern was observed for the aforementioned HARs related to genes *NPAS3* and *FZD8* that are active during CNS development in embryogenesis [51, 98].

Many studies were focused on finding differences between humans, chimpanzees and other mammals at the level of gene transcription [107–109]. Importantly, tissue-specific differences within the same species significantly exceeded in amplitude all species-specific differences in any tissue. The most transcriptionally divergent organs between humans and chimpanzees were liver and testis, and to a lesser extent – kidney and heart [107, 108]. A transcriptional distinction of liver may be a consequence of different nutritional adaptations in the two species. The major differences in testes are largely unexplained but may be related to predominantly monogamous behavior in humans. Surprisingly, the brain was the least divergent organ between humans and chimpanzees at the transcriptional level. In this regard, it is suggested that tighter regulation of signaling pathways in the brain underlies behavioral and cognitive differences [109, 110]. However, it was found that during evolution in the human cerebral cortex there were more transcriptional changes than in the chimpanzee [109]. Among them, the prevailed difference was increased transcriptional activity [110, 111]. In addition, many differences were identified in the alternative splicing patterns including 6–8% of gene exons, thus supporting a concept that the differentially spliced transcripts have pronounced functional consequences for the speciation [112].

Another study of transcriptional activity in the forebrain evidenced the higher difference between human and chimpanzee in the frontal lobe [113]. The functions of frontal lobe-specific groups of co-expressed genes dealt mostly with neurogenesis and cell adhesion [113]. Furthermore, the analysis of 230 genes associated with communication showed that about a quarter of them was differentially expressed in the brains of humans and other primates [110]. KRAB-zinc finger (*KRAB-ZNF*) genes were overrepresented among the genes differentially expressed in the brain [114]. Remarkably, the *KRAB-ZNF* gene family is known for its rapid evolution in primates, especially for its human- or chimpanzee-specific members [115]. The studies of transcriptional timing in the postnatal brain development also revealed a number of human-specific features. A specific set of genes was found whose expression was delayed in humans compared to the other primates. For example, the maximum expression of synaptic genes in the human prefrontal cortex was shifted from 1 year of age as for the chimpanzees and macaques, to 5 years. It is congruent with the prolonged brain development period in humans relative to other primates [116, 117]. The results recently published by Pollen and colleagues allowed to look deeper into the developing human and chimpanzee brains by applying the organoid model [118]. Cerebral organoids were generated from induced pluripotent stem cells (iPSCs) of humans and chimpanzees. Transcriptome analyses revealed 261 genes deferentially expressed in human versus chimpanzee cerebral organoids and macaque cortex. The PI3K/AKT/mTOR signaling axis appeared to be stronger activated in human, especially in radial glia [118].

Epigenetic regulation is another factor that should be considered when looking at interspecies differences in gene expression. High throughput analysis of differentially methylated DNA in human and chimpanzee brains showed that human promoters had lower degree of methylation. A fraction of genes related to neurologic/psychiatric disorders and cancer was enriched among the differentially methylated entries [118]. The analysis of H3K4me3 (trimethylated histone H3 is a marker of transcriptionally active chromatin) distribution in the neurons of prefrontal lobe revealed 471 human-specific regions, 33 of them were neuron-specific. Some of these regions were proximate to genes associated with neurologic and mental disorders, such as *ADCYAP1, CACNA1C, CHL1, CNTN4, DGCR6, DPP10, FOXP2, LMX1B, NOTCH4, PDE4DIP, SLC2A3, SORCS1, TRIB3, TUBB2B* and *ZNF423* [119, 120]. Another active chromatin biomarker is the distribution of DNase I hypersensitivity sites (DHSs), that often indicate gene regulatory elements. It was found that 542 DHSs overlapped with HARs, thus being so-called human accelerated DHSs, haDHSs [121]. Using chromatin immunoprecipitation assay, a number of haDHSs interacting genes were identified, many of which were connected with early development and neurogenesis [3, 121]. In a later study [122], about 3,5 thousand haDHSs were found, that were enriched near the genes related to neuronal functioning [122].

## Conclusions

It is now generally accepted that both changes in gene regulation and alterations of protein coding sequences might have played a major role in shaping the phenotypic differences between humans and chimpanzees. In this context, complex bioinformatic approaches combining various OMICS data analyses, are becoming the key for finding genetic elements that contributed to human evolution. It is also extremely important to have relevant experimental models to validate the candidate species-specific genomic alterations. The currently developing experimental methods such as obtaining pluripotent stem cells and target genome modifications, like CRISPR-CAS [105], open exciting perspectives for finding a “needle in haystack” that was truly important for human functional evolution, or probably many such needles. However, at least for now using these experimental approaches for millions of species specific potentially impactful features reviewed here is impossible due to high costs and labor intensity. In turn, an alternative approach could be combining the refined data in a realistic model of human-specific development using a new generation systems biology approach trained on a functional genomic Big Data of humans and other primates. Such an approach could integrate knowledge of protein-protein interactions, biochemical pathways, spatio-temporal epigenetic, transcriptomic and proteomic patterns as well as high throughput simulation of functional changes caused by altered protein structures. The differences revealed could be also analyzed in the context of mammalian and primate-specific evolutionary trends, e.g. by using dN/dS approach to measure evolutionary rates of structural changes in proteins [115] and enrichment by transposable elements in functional genomic loci to estimate regulatory evolution of genes [116]. Apart from the single-gene level of data analysis, this information could be aggregated to look at the whole organismic, developmental or intracellular processes e.g. by using Gene Ontology terms enrichment analysis [117] and quantitative analysis of molecular pathways [118].

And finally, most of the results described here were obtained for the human and chimpanzee reference genomes, which were built each using DNAs of several individuals. Nowadays the greater availability of whole-genome sequencing highlighted the next challenge in human and chimpanzee comparison – populational genome diversity. For example, the recent study [123] of 910 native African genomes was focused on the fraction of sequences absent from the reference Hg38 genome assembly. As many as 125,715 insertions missing in the Hg38 was identified with the average number of 859 insertions per individual, making up a total of 296,5 Mb. These findings clearly suggest that the current version of the human genome assembly can lack nearly 10% of the genome information. Furthermore, it also reflects the high degree of genome heterogeneity of the African population [123]. Similar studies were performed for other populations as well. For example, in the Chinese population a total of 29,5 Mb new DNA and 167 predicted novel genes missing in the reference genome assembly was discovered [124].

The chimpanzees also demonstrate substantial genome diversity with many population-specific traits: the central chimpanzees retain the highest diversity in the chimpanzee lineage, whereas the other subspecies show multiple signs of population bottlenecks [125].

So far there were not so many studies published on the topic of non-reference human and chimpanzee genome comparison. However, some estimates can be made. In the recent study of 1000 genomes from the Swedish population [126] there were identified totally 61,044 clusters totally making ~ 46 Mb of human DNA that were absent from the reference Hg38 human genome assembly. These clusters were called by the authors “new sequences” (NSs). As expected, NSs were enriched in simple repeats and satellites and varied greatly among the individuals. The most part of NSs (32,794) aligned confidently to the non-reference sequences from the aforementioned study of 910 African genomes [123]. Finally, as many as 18,773 NSs were present also in the chimpanzee PT4 genome assembly. In terms of protein coding sequences, 143 orthologous chimpanzee genes contained a total of 2807 NSs, where four genes were strongly enriched: EPPK1, OR8U1, NINL, and METTL21C. Positioning of NS insertions in the human genome revealed that 2195 of them located within 2384 genes, where 85 NS insertion events were found within the exons of 82 genes [126].

Another research consortium studied non-repetitive non-reference sequences (NRNR) in the genomes of 15,219 Icelanders [127]. A total of 326,596 bp of NRNR DNA was found, where ~ 84% was formed by only 244 insertions longer than 200 bp. Notably, comparison with the chimpanzee genome revealed that over 95% of the NRNRs longer than 200 bp were present also in the chimpanzee genome assembly, thus indicating that they were ancestral [127]. Thus, the lack of information on genome populational diversity could impact the total extent of human and chimpanzee interspecies divergence by misinterpretation of polymorphic sequences. However, it doesn’t abrogate most of the hypotheses and facts mentioned in this review. Still, these findings inevitably lead to the idea of the need, firstly, to create, and secondly, to compare human and chimpanzee pan-genomes.

## Data Availability

Not applicable.

## Abbreviations

Mya: Million years ago
Mb: Megabase (million base pairs)
kb: Kilobase (thousand base pairs)
HAR: Human accelerated region
HERV: Human endogenous retrovirus
LINE: Long interspersed nuclear element
PAR: Pseudoautosomal region
TE: Transposable element
